# Oxidative stress markers in tears of patients with Graves’ orbitopathy and their correlation with clinical activity score

**DOI:** 10.1186/s12886-018-0969-x

**Published:** 2018-11-21

**Authors:** Won Choi, Ying Li, Yong Sok Ji, Kyung Chul Yoon

**Affiliations:** Department of Ophthalmology and Research Institute of Medical Sciences, Chonnam National University Medical School and Hospital, 42 Jebong-ro, Dong-gu, Gwangju, 61469 South Korea

**Keywords:** Oxidative stress, Graves’ orbitopathy, Tear, 8-OHdG, MDA

## Abstract

**Background:**

To investigate the concentrations of oxidative stress markers, 8-hydroxy-2′-deoxyquanosine (8-OHdG) and malondialdehyde (MDA), in tears and their correlation with the clinical activity score (CAS) in patients with Graves’ orbitopathy (GO) according to disease activity.

**Methods:**

We recruited 27 participants with inactive stage GO, 35 participants with active stage GO, and 25 healthy controls without GO. The tear concentrations of 8-OHdG and MDA were determined by enzyme-linked immunosorbent assay. The correlation between CAS and the concentrations of tear 8-OHdG and MDA were analyzed according to the disease activity in the GO patients.

**Results:**

The levels of 8-OHdG and MDA were 56.30 ± 16.81 ng/mL and 5.39 ± 1.31 pmol/mg, respectively, in the control subjects, and 123.46 ± 22.67 ng/mL and 13.59 ± 3.93 pmol/mg, respectively, in patients with inactive stage GO, and 215.14 ± 35.61 ng/mL and 22.52 ± 4.63 pmol/mg, in patients with active stage GO. The mean concentrations of 8-OHdG and MDA were higher in patients with inactive and active stage GO compared with the control group (all *P* < 0.001). Furthermore, in the active stage group, tear concentrations of 8-OHdG and MDA were higher than those in the inactive stage group (all *P* < 0.001). The level of 8-OHdG (*r* = 0.676, P < 0.001) and MDA (*r* = 0.506, *P* = 0.002) correlated with CAS in the active stage GO group.

**Conclusions:**

The concentrations of 8-OHdG and MDA in tears increased in patients with GO, especially in those in the active stage. In patients with active stage GO, CAS correlated significantly with the tear 8-OHdG and MDA levels.

## Background

Thyroid-associated orbitopathy (eye disease), also known as Graves’ orbitopathy (GO) and, Graves’ ophthalmopathy is partly an autoimmune disease which can affect the periorbital and orbital tissues, and thyroid gland. Clinically detected GO occurs in 25 to 50% of patients with Graves’ disease (GD); nevertheless, severe form of GO can be developed only about 5% in patients with GD [1, 2]. GO is characterized by enlargement and inflammatory changes of the extraocular muscles, orbital connective and adipose tissues as a consequence of interactions between different autoantibodies and inflammatory mediators [3].

The pathogenesis of GO in GD considerably rests on the existence of an inflamed cell infiltrate mainly made up of activated T cells that, in turn, trigger secretion of glycosaminoglycans (GAG) by the activated orbital fibroblasts, further inducing orbital fibrosis and edema [4]. However, the detailed pathogenesis of GO is still unclear.

It has been recently reported that oxidative stress is associated with the pathogenesis of GO. Increased levels of extracellular reactive oxygen species (ROS) have been noted in the fibroadipose tissues, blood, orbital fibroblasts, and urine of GO patients [5–12]. Factors known to worsen oxidative stress, including cigarette smoking and ^131^I therapy, can also increase the prevalence of GO among GD patients [13, 14]. Cigarette smoking contains radicals and oxidants that cause systemic oxidative weight [15]. Therefore, it has been suggested that elevated ROS production by smoking overcomes oxidation reduction. In addition, Tsai et al. [6] have reveled oxidative deoxyribonucleic acid (DNA) destruction in the urine of GO patients and established a positive interrelation between clinical GO activity and this damage.

Moreover, in vivo and in vitro researches revealed that anti-oxidative parameters of oxidative stress and anti-thyroid drugs both in the whole organism and retro-orbital tissue [16]. In addition, a beneficial property of selenium has been established in patients with mild GO in double blind, placebo-controlled, multicenter clinical trials [17].

Until now, various markers of oxidative damage have been identified. Among these, 8-hydroxy-2′-deoxyquanosine (8-OHdG) and malondialdehyde (MDA) are the most commonly investigated by-products of DNA oxidation and lipid peroxidation [18]. 8-OHdG arises after the assault of the hydroxyl radical (the strongest ROS) at the guanine base in DNA [19]. MDA is generated from the mainly unsaturated fatty acids to their essential chains by oxidative mechanism [20].

To the best of our knowledge, no study has investigated the concentrations of oxidative stress markers in tears from patients with newly diagnosed GO, according to disease activity. The purpose of the current study is to analyze the levels of the oxidative stress markers such as 8-OHdG and MDA in tears according to the disease activity and to evaluate the correlation between these markers and the clinical activity score (CAS) in patients with inactive and active stage GO.

## Methods

### Subjects

Sixty-two patients with inceptively (< 2 months) diagnosed GD who were mostly consulted from the department of endocrinology and 25 healthy subjects without GD were enrolled in this study between January 2016 and December 2016. This study conducted in accordance with the tenets of the Declaration of Helsinki and performed with approval of the Chonnam National University Hospital institutional review board. Informed consent was acquired from every patients. A diagnosis of GD was made by endocrinologist on the basis of clinical signs and symptoms of thyrotoxicosis, laboratory findings of increased free thyroxine (T4) or triiodothyronine (T3) levels, and decreased thyroid-stimulating hormone levels and/or radiologic feature of diffuse homogeneous increased uptake in both lobes of the thyroid by ^99m^Technetium-pertechnetate scan. Every patients received complete ophthalmic examination including slit-lamp examination, refraction, visual acuity, intraocular pressure, ocular motility, fundus photography, Hertel exophthalmometry, palpebral fissure width, computed tomography, and thyroid hormone status including T3, T4, and free thyroxine (FT4). The diagnosis of GO was based on Bartley and Gorman’s criteria [21] that determine the clinical GO diagnosis in patients who have eyelid retraction in conjunction with minimum one of the following signs: proptosis, thyroid dysfunction, optic neuropathy or strabismus, after the elimination of other possible sources. In the cases of no eyelid retraction, the GO diagnosis can be considered even in the absence of other causal factors, if some signs mentioned above are in conjunction with thyroid dysfunction. Patients with a history of previous radioactive iodine therapy, thyroidectomy, any topical medication, ocular surgery or trauma, systemic drugs that may have toxicity of cornea, contact lenses, systemic diseases that can potentially affect the antioxidants levels such as diabetes mellitus, hypertension, coronary artery disease, alcoholism, or liver or kidney disorders, or any systemic disorder that can effect adversely the sub-basal nerve plexus was excluded. Control subjects were enrolled during routine screening visits and had no history of systemic disease, pathologic ocular findings, or ocular disease.

### Clinical activity score

The activity of inflammation in GO patients was assessed by the seven-point modification of the CAS, as previously described [22]. This CAS is based on classical signs and symptoms of inflammation (spontaneous pain, pain when moving the globe, redness of eyelids, redness of conjunctiva, swelling of plica/caruncle, swelling of eyelids, and chemosis). For each item present, one point is given. Each item has the same weight. The sum of these points is the CAS (range 0–7). A CAS ≥ 3/7 was indicated as active GO, and CAS < 3/7 was indicated as inactive GO.

### Tear collection and detection of 8-OHdG and MDA

As we previously described, basal tear secretions were harvested atraumatically from the both inferior tear meniscus using glass capillary tubes (Corning, Inc., Corning, NY) [23]. Caution was taken not to irritate the surfaces of the conjunctival and corneal. Fifty-microliter of tear materials were harvested and diluted by phosphate-buffered saline. Tear fluids were stored in microtubes and kept at − 70 °C until additional examination.

Total protein levels of the 8-OHdG (Cell Biolabs, San Diego, CA, USA) and MDA (Cell Biolabs) were detected using enzyme-linked immunosorbent assays (ELISA) in accordance with the producer’s instructions [24]. Tear specimens were centrifuged at 3000 g during 10 min or filtered through 0.45 mm filter, prior to use in the ELISA. Both 8-OHdG and MDA protein samples (50 μl) were absorbed onto the 8-OHdG and MDA conjugate coated plate, respectively. The level of 8-OHdG and MDA in an unknown sample was measured by comparing its absorption with the known standard curve. The minimal detectable concentrations of the 8-OHdG and MDA were above 0.078 ng/mL and 6 pmol/mg, respectively.

### Statistical analysis

SPSS version 18.0 (SPSS, Chicago, IL) was employed for all statistical analyses. All data are presented as mean ± standard deviation. Groups were compared using the Chi-square, independent *t* test, and one-way analysis of variance with post hoc test for comparing results between groups; Pearson correlation coefficients were assessed for the correlations between CAS and thyroid hormone status on the one hand and tear 8-OHdG and MDA levels on the other hand. Receiver operator curves (ROCs) were created for analyzing the sensitivity and specificity of active stage GO. A *P* value of less than 0.05 was considered statistically significant.

## Results

### Patient clinical features and demographics

Patient clinical features and demographics in the three groups are presented in Table 1. There were 25 patients (9 men and 16 women) in control participants, 27 patients (7 men and 20 women) in the inactive stage group, and 35 patients (13 men and 22 women) in the active stage group. Mean subject age was 38.56 ± 11.78 years (range: 21–59 years), 36.56 ± 13.75 years (range: 20–65 years), and 41.48 ± 14.87 years (range: 20–66 years) in the control and inactive and active stage groups, respectively. There were no statistically significant differences in sex, age, visual acuity, proptosis, palpebral fissure width, and thyroid hormone state (*P* > 0.05). However, the CAS (*P* = 0.001) and extraocular muscle enlargement in orbital computed tomography (*P* = 0.035) showed significant differences in the active stage group compared to that in the inactive stage group.

**Table 1:** Patient demographics and clinical features of the control subjects and patients with inactive and active stages of Graves’ orbitopathy

	Control (*n* = 25)	Inactive stage (*n* = 27)	Active stage (*n* = 35)	*P* value
Sex (M/F)	9/16	7/20	13/22	0.674
Mean age (years)	38.56 ± 11.78	36.56 ± 13.75	41.48 ± 14.87	0.153
Visual acuity (LogMAR)	0.03 ± 0.02	0.04 ± 0.02	0.10 ± 0.14	0.238
Proptosis (mm)	16.12 ± 4.83	18.11 ± 3.38	17.54 ± 5.29	0.513
Palpebral fissure width (mm)	12.28 ± 2.54	11.26 ± 3.38	12.49 ± 3.74	0.911
Clinical activity score		1.33 ± 0.62	4.34 ± 1.30	0.001
EOM enlargement in orbital CT		10 (37.0%)	19 (54.3%)	0.035
Thyroid hormone state				
Hyperthyroid state		16 (59.3%)	20 (57.1%)	0.781
Euthyroid state		10 (37.0%)	13 (40.0%)	0.684
Hypothyroid state		1 (3.7%)	2 (5.7%)	0.305

*EOM* Extraocular muscle, *CT* Computed tomography

### Tear 8-OHdG and MDA levels and their correlation with CAS

**Fig. 1:**
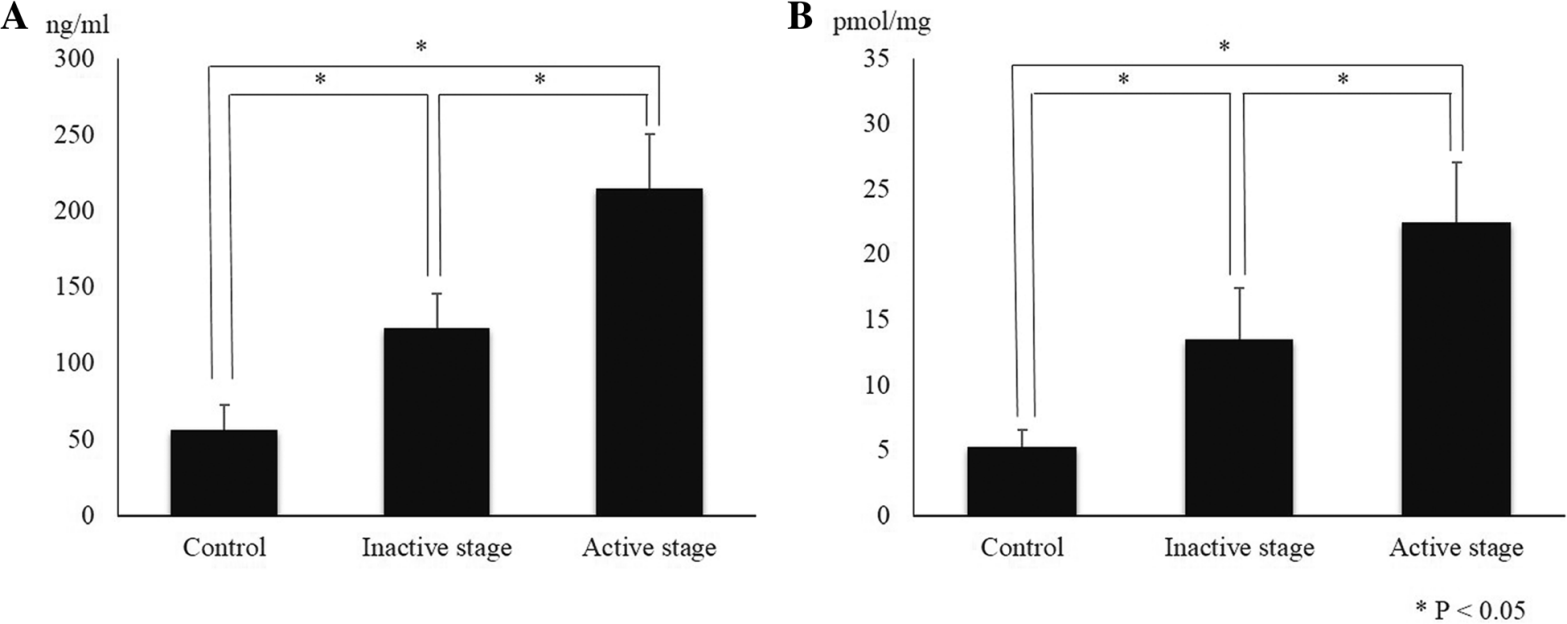
8-OHdG (**a**) and MDA (**b**) levels in tears of the control subjects and patients with inactive and active stage Graves’ orbitopathy

The mean concentrations of 8-OHdG and MDA were 56.30 ± 16.81 ng/mL and 5.39 ± 1.31 pmol/mL in control subjects, and 123.46 ± 22.67 ng/mL and 13.59 ± 3.93 pmol/mg in inactive stage patients, and 215.14 ± 35.61 ng/mL and 22.52 ± 4.63 pmol/mg, in active stage patients, respectively. The mean levels of 8-OHdG and MDA showed significant differences in the inactive and active stage groups than in the control group (all *P* < 0.001). Moreover, in the active stage GO group, 8-OHdG and MDA levels in tear fluid showed significantly higher results than those in the inactive stage GO group (all P < 0.001) (Fig. 1).

**Fig. 2:**
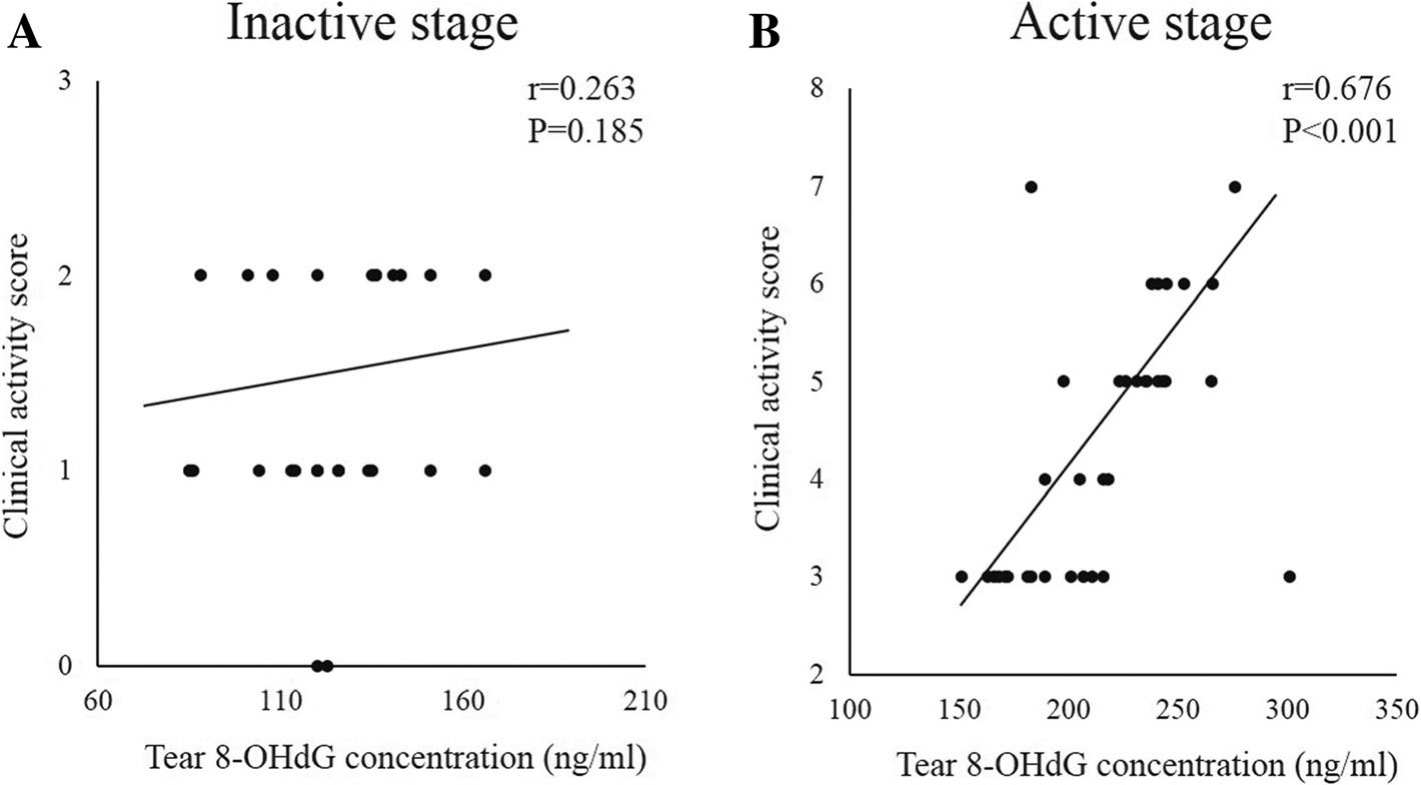
Correlations between the tear 8-OHdG concentrations and clinical activity score of the patients with inactive stage (**a**) and active stage (**b**) Graves’ orbitopathy

**Fig. 3:**
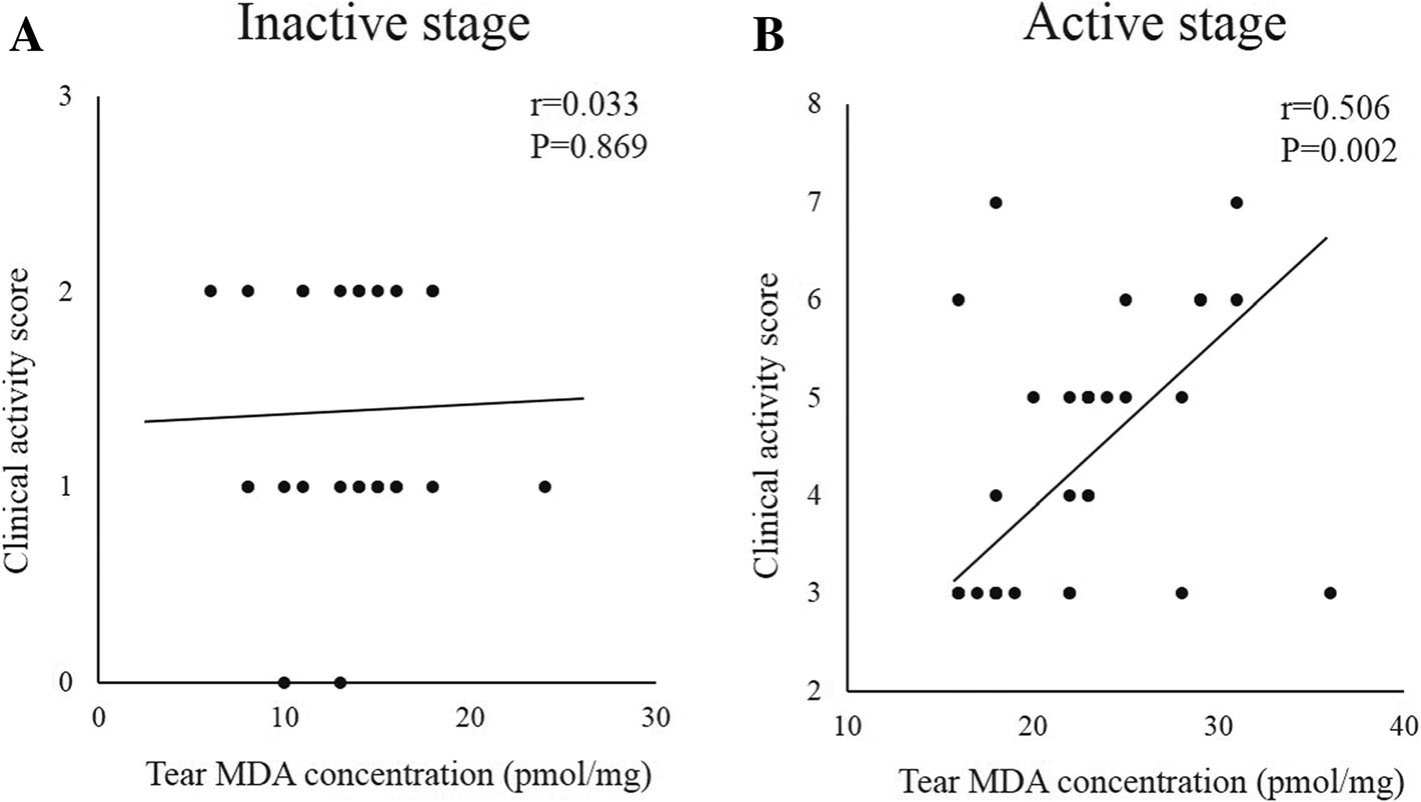
Correlations between the tear MDA concentrations and clinical activity score of the patients with inactive stage (**a**) and active stage (**b**) Graves’ orbitopathy

In the inactive stage GO patients, the correlations between the CAS and tear 8-OHdG (*r* = 0.263, *P* = 0.185) and MDA (*r* = 0.033, *P* = 0.869) concentrations showed no significant results. On the other hand, in the active stage group, the tear 8-OHdG (*r* = 0.676, *P* < 0.001) and MDA (*r* = 0.506, *P* = 0.002) levels correlated significantly with the CAS (Figs. 2 and 3). In the GO patients, the correlation between tear 8-OHdG and MDA levels in each subject showed significant result (*r* = 0.667, *P* < 0.001).

The cut-off value of the tear 8-OHdG and MDA level were defined as 186 ng/mL and 19.50 pmol/mL. From the ROC curves, these concentrations to predict active stage GO showed 95.2% sensitivity and 85.4% specificity in the 8-OHdG level and 85.7% sensitivity and 87.8% specificity in the MDA level, respectively.

In addition, correlations between tear 8-OHdG levels and serum T3 (*r* = 0.104, *P* = 0.381), T4 (*r* = 0.169, *P* = 0.290), FT4 (*r* = 0.255, *P* = 0.213) status showed no significant results. Similar results were obtained between tear MDA levels and serum T3 (*r* = 0.127, *P* = 0.341), T4 (*r* = 0.037, *P* = 0.791), FT4 (*r* = 0.139, *P* = 0.322) status.

## Discussion

The major findings of the present study were as follows. First, compared with the control, increased levels of 8-OHdG and MDA in the tear film were observed in patients with GO, especially with active stage. Second, the levels of the 8-OHdG and MDA were positively correlated with CAS and reflect the disease severity in the active stage GO group. The present study offers interesting and possibly important findings on the role of 8-OHdG and MDA in the pathogenesis of GO. To the best of our knowledge, this is the first report of increased oxidative stress marker levels in the tear fluid and their correlation with the CAS in GO patients according to disease activity. Our data support the role of oxidative stress in development of GO in accordance with previous findings.

Both 8-OHdG and MDA, in addition to hydrogen peroxide and intracellular superoxide anion, were considerably increased in GO orbital fibroblasts compared with normal controls [5]. The study by Tsai et al. [6] established that the level of 8-OHdG in urine was considerably elevated in GO patients (1.9-fold in comparison with normal subjects). This elevation was remarkable in active GO patients (2.4-fold in comparison with normal subjects). Furthermore, 8-OhdG levels in the urine significantly correlated with the TSH receptor antibody levels, CAS, and ophthalmopathy index. They also reported in another study that long term stress-induced ROS overproduction was due to the increase of manganese-dependent superoxide dismutase activity and concomitant decrease of glutathione peroxidase (GPx) activity, resulting in increased accumulation of hydrogen peroxide in GO orbital fibroblasts [25]. Moreover, the prominent decrease of glutathione/oxidized glutathione ratio and the diminished GPx activity in GO orbital fibroblasts indicated a significant redox imbalance in the cells, that in turn led to additional aggregation of endogenous hydrogen peroxide in the orbital fibroblasts with GO patients [25].

ROS (superoxide anions and hydrogen peroxide) trigger the production of pro-inflammatory cytokines that play a pivotal role in the GO development and accelerate proliferation of fibroblast in the orbit by dose-dependent way; the proliferative effect can be decreased with several antioxidants and methimazole [26, 27]. IL-1β induces free radical generation in the fibroblasts of GO and normal control, and enhances superoxide dismutase activity in GO orbital fibroblasts. Moreover, decreasing oxygen-free radicals with superoxide dismutase and catalase partially inhibited GAG production triggered by IL-1β [11].

Based on this mechanism, there have been several articles that antioxidants have a promising role in the prevention of GO progression at mild to moderately active GO. In the first pilot antioxidant supplementation study, allopurinol and nicotinamide therapy decreased total eye score and soft tissue swelling and improved patients’ satisfaction in 82% of participants with mild to moderately severe GO [28]. Selenium is integrated into several selenoproteins and acts as an antioxidant, decreases thyroperoxidase antibodies in patients with autoimmune thyroiditis [29]. A following randomized, double - blind, controlled trial of selenium ingestion for 6 months in GO patients led to an improved appearance, decreased soft tissue inflammation, improved quality of life, and delayed progression of GO compared to the control group [17]. These results indicate increased lipid peroxidation and oxidative DNA damage can play an pivotal role in the pathogenesis of GO.

Several articles report that the tear fluid and lacrimal gland can be directly accompanied in the damage of the ocular surface in GO patients [30–33]. However, the exact mechanisms of increased oxidative stress markers in the tears with GO have not been appropriately explained. We put forward the following hypothesis. Initially, lacrimal glands have been described as the general target organs for the thyroid hormones [34]. This might lead to the binding of autoantibodies to thyrotropin receptor, and antigen-specific response can arouse oxidation and deteriorate the function of lacrimal gland. Finally, the affected lacrimal gland may induce ROS and oxidative markers that are secreted into tears. Therefore, the component of oxidative stress markers in the tears can be altered.

As described above, studies conducted to date about the pathogenesis of GO have focused mostly on orbital tissues obtained by surgery. The disadvantages of this method are that it is invasive, difficult to obtain permission from every patient, and only available from patients through surgery. On the other hand, tear collection is a relatively non-invasive technique used to take samples from the ocular surface to determine oxidative stress markers in GO patients. The advantage of tear sampling method is that it is easy to get permission from most patients, triggers less pain and discomfort, and can be measured repeatedly as time passes.

## Conclusions

In summary, we firstly demonstrated the tear 8-OHdG and MDA levels and their correlation with the CAS in patients with GO. The tear 8-OHdG and MDA levels may be used as potential adjuvant biomarkers in the determination of disease activity in patients with active stage GO. In addition, these markers may also be useful for distinguishing between active and inactive stage GO. Modulation of these oxidative stress markers could be considered as a future therapeutic approach for GO.

## Abbreviations

8-OHdG: 8-hydroxy-2′-deoxyquanosine
DNA: Deoxyribonucleic acid
GAG: Glycosaminoglycans
GD: Graves’ disease
GO: Graves’ orbitopathy
GPx: Glutathione peroxidase
MDA: Malondialdehyde
ROCs: Receiver operator curves
ROS: Reactive oxygen species

## References

[1] Abraham-Nordling M, Bystrom K, Torring O, Lantz M, Berg G, Calissendorff J (2011). Incidence of hyperthyroidism in Sweden. Eur J Endocrinol.

[2] Garrity JA, Bahn RS (2006). Pathogenesis of graves ophthalmopathy: implications for prediction, prevention, and treatment. Am J Ophthalmol.

[3] Bahn RS (2015). Current insights into the pathogenesis of Graves’ Ophthalmopathy. Horm Metab Res.

[4] Weetman AP (2000). Graves’ disease. N Engl J Med.

[5] Tsai CC, Wu SB, Cheng CY, Kao SC, Kau HC, Chiou SH (2010). Increased oxidative DNA damage, lipid peroxidation, and reactive oxygen species in cultured orbital fibroblasts from patients with Graves’ ophthalmopathy: evidence that oxidative stress has a role in this disorder. Eye (Lond).

[6] Tsai CC, Cheng CY, Liu CY, Kao SC, Kau HC, Hsu WM (2009). Oxidative stress in patients with Graves’ ophthalmopathy: relationship between oxidative DNA damage and clinical evolution. Eye (Lond).

[7] Hondur A, Konuk O, Dincel AS, Bilgihan A, Unal M, Hasanreisoglu B (2008). Oxidative stress and antioxidant activity in orbital fibroadipose tissue in Graves’ ophthalmopathy. Curr Eye Res.

[8] Bednarek J, Wysocki H, Sowinski J (2005). Oxidative stress peripheral parameters in Graves’ disease: the effect of methimazole treatment in patients with and without infiltrative ophthalmopathy. Clin Biochem.

[9] Tsai CC, Kao SC, Cheng CY, Kau HC, Hsu WM, Lee CF (2007). Oxidative stress change by systemic corticosteroid treatment among patients having active graves ophthalmopathy. Arch Ophthalmol.

[10] Bednarek J, Wysocki H, Sowinski J (2004). Peripheral parameters of oxidative stress in patients with infiltrative Graves’ ophthalmopathy treated with corticosteroids. Immunol Lett.

[11] Lu R, Wang P, Wartofsky L, Sutton BD, Zweier JL, Bahn RS (1999). Oxygen free radicals in interleukin-1beta-induced glycosaminoglycan production by retro-ocular fibroblasts from normal subjects and Graves’ ophthalmopathy patients. Thyroid.

[12] Bartalena L, Tanda ML, Piantanida E, Lai A (2003). Oxidative stress and Graves’ ophthalmopathy: in vitro studies and therapeutic implications. Biofactors.

[13] Hegedius L, Brix TH, Vestergaard P (2004). Relationship between cigarette smoking and Graves’ ophthalmopathy. J Endocrinol Investig.

[14] Bartalena L, Marcocci C, Bogazzi F, Manetti L, Tanda ML, Dell’Unto E (1998). Relation between therapy for hyperthyroidism and the course of Graves’ ophthalmopathy. N Engl J Med.

[15] Pryor WA, Stone K (1993). Oxidants in cigarette smoke. Radicals, hydrogen peroxide, peroxynitrate, and peroxynitrite. Ann N Y Acad Sci.

[16] Zarkovic M (2012). The role of oxidative stress on the pathogenesis of graves’ disease. J Thyroid Res.

[17] Marcocci C, Kahaly GJ, Krassas GE, Bartalena L, Prummel M, Stahl M (2011). Selenium and the course of mild Graves’ orbitopathy. N Engl J Med.

[18] Chang YT, Chang WN, Tsai NW, Huang CC, Kung CT, Su YJ (2014). The roles of biomarkers of oxidative stress and antioxidant in Alzheimer's disease: a systematic review. Biomed Res Int.

[19] Karihtala P, Kauppila S, Puistola U, Jukkola-Vuorinen A (2011). Divergent behaviour of oxidative stress markers 8-hydroxydeoxyguanosine (8-OHdG) and 4-hydroxy-2-nonenal (HNE) in breast carcinogenesis. Histopathology.

[20] Ma Y, Zhang L, Rong S, Qu H, Zhang Y, Chang D (2013). Relation between gastric cancer and protein oxidation, DNA damage, and lipid peroxidation. Oxidative Med Cell Longev.

[21] Bartley GB, Gorman CA (1995). Diagnostic criteria for Graves’ ophthalmopathy. Am J Ophthalmol.

[22] Mourits MP, Prummel MF, Wiersinga WM, Koornneef L (1997). Clinical activity score as a guide in the management of patients with Graves’ ophthalmopathy. Clin Endocrinol.

[23] Choi W, Li Z, Oh HJ, Im SK, Lee SH, Park SH (2012). Expression of CCR5 and its ligands CCL3, −4, and −5 in the tear film and ocular surface of patients with dry eye disease. Curr Eye Res.

[24] Choi W, Lian C, Ying L, Kim GE, You IC, Park SH (2016). Expression of lipid peroxidation markers in the tear film and ocular surface of patients with non-Sjogren syndrome: potential biomarkers for dry eye disease. Curr Eye Res.

[25] Tsai CC, Wu SB, Cheng CY, Kao SC, Kau HC, Lee SM (2011). Increased response to oxidative stress challenge in Graves’ ophthalmopathy orbital fibroblasts. Mol Vis.

[26] Tsai CC, Wu SB, Kao SC, Kau HC, Lee FL, Wei YH (2013). The protective effect of antioxidants on orbital fibroblasts from patients with Graves’ ophthalmopathy in response to oxidative stress. Mol Vis.

[27] Burch HB, Lahiri S, Bahn RS, Barnes S (1997). Superoxide radical production stimulates retroocular fibroblast proliferation in Graves’ ophthalmopathy. Exp Eye Res.

[28] Bouzas EA, Karadimas P, Mastorakos G, Koutras DA (2000). Antioxidant agents in the treatment of Graves’ ophthalmopathy. Am J Ophthalmol.

[29] Duntas LH, Mantzou E, Koutras DA (2003). Effects of a six month treatment with selenomethionine in patients with autoimmune thyroiditis. Eur J Endocrinol.

[30] Moncayo R, Baldissera I, Decristoforo C, Kendler D, Donnemiller E (1997). Evaluation of immunological mechanisms mediating thyroid-associated ophthalmopathy by radionuclide imaging using the somatostatin analog 111In-octreotide. Thyroid.

[31] Khalil HA, de Keizer RJ, Kijlstra A (1988). Analysis of tear proteins in Graves’ ophthalmopathy by high performance liquid chromatography. Am J Ophthalmol.

[32] Matheis N, Grus FH, Breitenfeld M, Knych I, Funke S, Pitz S (2015). Proteomics differentiate between thyroid-associated Orbitopathy and dry eye syndrome. Invest Ophthalmol Vis Sci.

[33] Matheis N, Okrojek R, Grus FH, Kahaly GJ (2012). Proteomics of tear fluid in thyroid-associated orbitopathy. Thyroid.

[34] Dias AC, Modulo CM, Jorge AG, Braz AM, Jordao AA, Filho RB (2007). Influence of thyroid hormone on thyroid hormone receptor beta-1 expression and lacrimal gland and ocular surface morphology. Invest Ophthalmol Vis Sci.

